# Association of thyroid hormones with resting energy expenditure and complement C3 in normal weight high body fat women

**DOI:** 10.1186/s13044-019-0070-4

**Published:** 2019-10-25

**Authors:** Maryam Karkhaneh, Mostafa Qorbani, Asal Ataie-Jafari, Mohamad Reza Mohajeri-Tehrani, Hamid Asayesh, Saeed Hosseini

**Affiliations:** 1 Department of Community Nutrition, School of Nutritional Sciences and Dietetics, Tehran University of Medical Sciences, Tehran, Iran; 2 Non-communicable Diseases Research Center, Alborz University of Medical Sciences, Karaj, Iran; 3 Chronic Diseases Research Center, Endocrinology and Metabolism Population Sciences Institute, Tehran University of Medical Sciences, Tehran, Iran; 4 Department of Nutrition, Faculty of Medicine, Science and Research Branch, Islamic Azad University, Tehran, Iran; 5 Endocrinology and Metabolism Research Center, Endocrinology and Metabolism Clinical Sciences Institute, Tehran University of Medical Sciences, Tehran, Iran; 6 Department of Medical Emergencies, Qom University of Medical Sciences, Qom, Iran; 7 Department of Clinical Nutrition, School of Nutritional Sciences and Dietetics, Tehran University of Medical Sciences, Hojatdost street, Naderi street, Keshavarz Blv, Tehran, Iran

**Keywords:** Normal Weight Obesity, Body fat mass, Triiodothyronine, Thyroxine, Complement C3, Resting energy expenditure

## Abstract

**Background:**

A high body fat percentage has a specific effect on activation of the hypothalamic-pituitary-thyroid axis. On the other hand, a slight change in thyroid hormones can affect metabolism and body composition as well as immune function. This study aims to examine the relationship between adiposity, thyroid hormone levels and immunity by comparing resting energy expenditure (REE), serum thyroid hormone levels and complement C3 in normal-weight high body fat (normal weight obesity) women and normal-weight normal body fat women.

**Methods:**

In this case-control study, 40 women with normal body weight (BMI < 24.9 kg/m2) and body fat mass (FM) ≥ 30% (normal-weight obesity (NWO) group), and 30 non-obese women (BMI < 24.9 kg/m2) and FM < 30% (non-normal weight obesity (non-NWO) group) were recruited from a sport club in Tehran. Body composition was analyzed using bioimpedance analyzer. Blood samples were collected and analyzed for fasting serum concentration of thyroid hormones (including total T3 and total T4), thyroid-stimulating hormone (TSH), and serum complement C3. REE was measured by an indirect calorimetry.

**Results:**

Serum T3 and T4 and also complement C3 were higher in the NWO group than in the non-NWO group. Body fat percentages had significant positive correlation with T3 (r; 0.344, *P* <  0.05), T4 (r; 0.294, *P* <  0.05), and complement C3 (r; 0.417, P <  0.05). Serum T3 and T4 were also positively correlated with C3 concentration (r; 0.417, *p* <  0.001) and (r; 0.349, *p* <  0.05); respectively, but there was no significant correlation between TSH and C3. REE was not significantly different between the two groups. REE only had a significant positive correlation with fat-free mass (r; 0.421, *P* <  0.001).

**Conclusion:**

An increase in body fat even in the presence of a normal body weight can be accompanied by the changes in thyroid function and inflammatory markers such as complement C3.

## Introduction

Excess adiposity has been associated with pathophysiologic processes, including insulin resistance, altered lipid metabolism, metabolic disorders and endothelial dysfunction [1–3]. Even subjects who have normal body weight [based on body mass index (BMI)] but high body fat mass (FM) content [normal weight obesity (NWO)] are at higher risk for cardiometabolic dysregulation and cardiovascular diseases compared to subjects with normal weight and lower FM (within the normal range) [3–6].

Accumulation of fat and development of adipose tissue activate the hypothalamic-pituitary-thyroid (HPT) axis (by cytokines) causing changes in thyroid function [7–9]. On the other hand, the thyroid hormones are a major determinant of energy metabolism, and defects in thyroid function are associated with variations in body weight and body composition [10–14]. Evidence suggests that small changes in thyroid hormone levels, even within reference ranges, may contribute to increasing of regional adiposity and a tendency to gain weight [8, 15, 16]; however, this has not been confirmed by all studies [17].

Thyroid dysfunction is not only associated with changes in body composition, body temperature, and total resting energy expenditure independent of physical activity [8, 9, 18, 19], thyroid gland also has a direct mutual relationship with the immune system [20–23]. Thyroid hormones can affect complement C3 production, its conversion to Acylation stimulating protein (ASP) and other markers of inflammation and immunity [20, 24, 25]. Triiodothyronine (T3) is capable of modifying immune function via nuclear receptors (TRs) to regulate target genes, and by non-genomic interactions with membrane receptors independent of protein synthesis [20, 26]. Moreover, higher T4 concentrations are significantly associated with higher concentrations of the complement proteins C3 and C4 [20].

Complement C3, a major component of human innate immunity, can be released by adipose tissue and liver. In addition to its role in the generation of ASP, C3 appears to be linked to insulin resistance and concentrations of plasma lipids directly, an association that is specific for this complement protein. C3 correlates strongly with insulin resistance and metabolic syndrome [3, 27–29] and can affect resting metabolic rate and thyroid function [30–32].

In order to examine the complex relationship between adiposity, thyroid hormone levels and immunity, this study aimed to compare serum thyroid hormone levels and their association with fat mass (FM), resting energy expenditure (REE), and C3 in normal-weight high FM women and normal-weight lower FM (within the normal range) women.

## Methods

### Subjects

In this case-control study, 70 normal-weight Iranian women were divided into two groups: (1) 40 women with BMI < 24.9 kg/m^2^ and FM ≥ 30% (NWO group), and (2) 30 age-matched controls with BMI < 24.9 kg/m^2^ and FM < 30% (non-NWO group).

The subjects were recruited by announcements in the youth club of Tehran. Women aged 19–39 years with normal BMI who had recently joined the club were selected to measure body composition using the bioelectric impedance analyzer (BIA).

The study exclusion criteria included known diabetes, hypertension, dyslipidemia, liver diseases, kidney diseases, cardiovascular diseases, Cushing’s syndrome, thyroid diseases, autoimmune diseases, lactation, and pregnancy. Almost all of the women were in the follicular phase of the menstrual cycle and did not smoke or abuse alcohol.

After reading and signing the written consent form, each participant was invited to receive a series of tests. A fasting blood sample was drawn between 8:00 and 9:00 am for determination of fasting serum complement C3, thyroid hormones (total T3 and total T4), and TSH. Body composition and REE were measured in the fasting state.

### Measurements

Height was measured without shoes using a digital meter mounted on a wall to the nearest 0.5 cm. Weight was measured by the TANITA body composition analyzer in fasting condition with minimal clothes and without shoes, after defecation, at 8 to 9 am. BMI was calculated by dividing weight (in kilograms) by height squared (in meters), which was automatically performed by the TANITA analyzer.

Analysis of body composition was carried out using the BIOIMPEDANCE method and TANITA Body Composition Analyzer (BC-418MA) in specific conditions, i.e. fasting, not drinking too much water, and avoiding physical activity and exercise before the test, between 8 and 9 o’clock in the morning.

REE was determined in the morning after an overnight fast using an indirect calorimetry (META CHECK).

All participants were asked to fill out a METs questionnaire (Metabolic Equivalents) for the analysis of physical activity.

### Blood samples

The venous blood samples (10 ml) were collected after a 10–12 h overnight fast and serum concentrations of complement C3, thyroid hormones including total T3 and total T4 (normal range:75–200 ng/dl for T3 and 5.5–12.5 μg/dl for T4), and TSH measured. Two ml of blood samples were transferred to sterile tubes containing anticoagulant EDTA, and the remaining 8 ml was poured into other vacuum tubes for faster coagulation. All materials were immediately placed in ice. Serum was obtained by centrifugation at 1500 rpm for 10 min. All samples were stored at − 80 °C and analyzed after 3–4 months. Serum complement C3 was measured by the 902 Hitachi autoanalyzer through the photometric method using Pars test kits and serum thyroid hormones concentration was determined by the ELISA method using the Monobind kit.

### Statistical analyses

The data were analyzed using SPSS software (version 16.0; SPSS Inc., Chicago, IL, USA). Normal distribution of continuous variables was checked by Kolmogorov-Smirnov test. Continuous variables with normal distribution are reported as the mean ± standard deviation (SD). Since BMI was statistically different between NWO and non-NWO, the adjusted mean of thyroid hormones, inflammatory markers and REE between the groups was compared using analysis of covariance (ANCOVA) test. The partial Pearson’s correlation coefficient (adjusted for BMI) was used to assess the correlation between continuous variables. *P*-value < 0.05 was considered as statistically significant.

## Results

### Participant characteristics

Participants’ characteristics are summarized in Table 1. Mean age (SD) in NWO and non-NWO groups was similar. There were significant differences in weight, hip circumference (HC), waist circumference (WC), BMI and FM between the two groups. Physical activity levels, height and WC to HC ratio were not significantly different between the groups.

**Table 1:** Mean and standard deviation of the participants’ characteristics in NWO and non-NWO

	NWO (*n* = 40)	Non-NWO (*n* = 30)	*P* value
	Mean ± SD	Mean ± SD	
Age (y)	28.45 ± 4.87	28.83 ± 4.39	0.735
H (cm)	166.12 ± 4.45	165.68 ± 4.72	0.690
W (kg)	62.97 ± 4.92	57.14 ± 4.20	< 0.001
BMI (kg/m^2^)	22.67 ± 1.26	20.85 ± 1.32	< 0.001
WC (cm)	74.79 ± 4.82	70.77 ± 2.91	< 0.001
HC (cm)	99.13 ± 4.32	93.17 ± 2.91	< 0.001
WC/HC	0.75 ± 0.04	0.76 ± 0.03	0.448
FM (%)	32.75 ± 2.62	23.52 ± 1.68	< 0.001
FM (kg)	20.60 ± 2.92	13.41 ± 1.43	< 0.001
FFM (KG)	42.12 ± 2.83	43.52 ± 3.16	0.057
MET_S_	37.35 ± 3.55	38.61 ± 2.5	0.103

*NWO* Normal weight obesity, *H* Height, *W* Weight, *BMI* Body mass index, *WC* Waist circumference, *HC* Hip circumference, *W/H* Waist to hip ratio, *FFM* Fat-free mass, *FM* Fat mass, *MET* Metabolic Equivalents

### Comparison of resting energy expenditure and serum thyroid hormone levels with complement C3 levels in the NWO and the non-NWO groups

Adjusted mean of resting energy expenditure, resting energy expenditure per kilogram of fat-free mass and biochemical parameters, including serum thyroid hormones (total T3 and total T4), TSH and complement C3 are shown in

**Table 2:** Adjusted mean and standard deviation of resting energy expenditure, thyroid hormones (total T3 and total T4) and inflammatory markers in NWO and non-NWO

	NWO (*n* = 40)		Non-NWO (*n* = 30)		*P*-value^*^
REE (kcal/day)	1348 ±	156.6	1338 ± 92.6		0.730
REE/ FFM (kg)	31.88 ±	3.24	30.96 ± 2.20		0.116
T3 (ng/dl)	103.64	± 19.77	92.36 ± 12.05		0.002
T4 (μg/dl)	8.37 ± 1.09		7.68	± 0.99	0.006
TSH (mIU/L)	2.19 ± 1.16		2.70 ± 1.04		0.079
C3 (g/l)	104.30 ± 15.04		92.79	± 8.13	< 0.001

REE: resting energy expenditure *Adjusted for BMI based on ANCOVA test

C3: serum Complement C3, T3: triiodothyronine, T4: Thyroxine

*Adjusted for BMI based on ANCOVA test

Table 2**.** There were no statistically significant differences between the two groups in REE and REE per kilogram of fat-free mass. Serum T3, T4, and complement C3 were significantly higher in NWO group compared to non- NWO, however, these measurements were within the normal range. Also there was no significant different in serum TSH between the two groups.

### Correlations between body fat mass and resting energy expenditure, serum thyroid hormone levels and complement C3

There was no significant correlation between body fat mass and REE (r − 0.066). REE only had a significant positive correlation with fat-free mass (r 0.421, *P* <  0.001).

Body fat percentages had significant positive correlation with T3 (r 0.344, *P* <  0.05) and T4 (r 0.294, P <  0.05). There was no significant correlation between body fat percentage and serum TSH (*P* > 0.05).

There was significant correlation between body fat percentages and complement C3 (r 0.417, P <  0.05).

After adjustment for C3 concentration, a positive significant correlation was also found between REE and thyroid hormones (r; 0.265, P <  0.05). Additionally, the negative correlation between REE and C3 was not statistically significant (r − 0.21).

Serum T3 and T4 were positively correlated with C3 concentration (r 0.417, *p* <  0.001) and (r 0.349, *p* <  0.05), respectively; but there was no significant correlation between TSH and C3.

## Discussion

### Resting energy expenditure and complement C3 in normal weight obesity

In this study, REE was not different between the NWO and non-NWO groups, even after adjustment for fat-free mass. There was a significant correlation between REE and fat-free mass. Few studies have measured REE in NWO subjects. The study of Conus et al. [33] confirms the findings of our study, while Di Renzo et al. [34] demonstrated lower REE in NWO in comparison with nonobese women. A major determinant of REE is body composition, specifically metabolically active tissues, fat-free mass, with lower influences of fat mass and other factors [35]. Some studies have shown that REE is significantly higher among the obese than the non-obese [36, 37]. Although, it has been shown that energy expenditure is actually lower in relation to weight in overweight and obese people with high fat mass [38].

On the other hand, REE, as the most important component of energy consumption, can predict the development of obesity [39].

In our study the concentration of serum C3 was higher in subjects with higher fat percentages (NWO), than the control non-NWO group (this increase was within the normal range) and there was no significant correlation between serum C3 and REE in the NWO group. In line with our study, findings from previous studies suggest a relationship between adiposity and systemic C3. This link is verified by the observation that the adipose tissue secretes C3 [40].

On the other hand, in contrast to our study, the study of Xia et al. [41] showed a significant and negative relationship between serum C3 and REE in obese mice. An increase in body fat mass and serum complement C3 can reduce REE. The mechanism was explained as elimination of C3 (ASP precursor) and ASP reduction, resulting in reduced fat mass, increased oxygen consumption, and increased fatty acid oxidation in the liver and muscle of mice, by increasing the expression of the mRNA related to uncoupling proteins 2 and 3 (UCP2 and UCP3) [41]. C3 can stimulate lipogenesis and triglyceride synthesis in adipocytes; and preventing the release of free fatty acids derived from lipolysis [27].

### Thyroid hormones and resting energy expenditure in normal weight obesity

Our study showed that levels of thyroid hormones (total T3 and total T4) had a significant positive correlation with body fat percentage, and were significantly higher in NWO group compared to non-NWO (this increase was within the normal range). However, TSH levels were not statistically different between the two groups. In addition, there was a significant positive correlation between resting energy expenditure and thyroid hormones after controlling for the effect of complement C3. The findings of our study showing high levels of thyroid hormones in the NWO group and significant positive correlation between thyroid hormones with body fat percentage and resting energy expenditure were in line with the findings from the previous studies [8, 19, 42–44]. In contrast to our study, some previous studies observed a slight increase in TSH levels in obese subjects [42–44].

An adaptive mechanism in response to an increase in body fat mass and its secreted cytokines, along pathways affecting the HPT axis, results in a slight increase in the thyroid stimulating hormone and thyroid hormones [42, 43]. Several studies suggest the impact of leptin secreted from extra fat mass on TSH release (via weight-affecting hormones and neurotransmitters), increased deiodinase activity, and increased conversion of T4 to T3 [44]. The findings of some studies indicate that T3 modulates the gene expression, serum levels of leptin, resistin, and adiponectin in obesity [45, 46]. TSH also increases leptin production through its receptors in adipose tissue. Although thyroid function is usually normal in obese subjects, many studies have shown that levels of TSH and sometimes T3 increases slightly [44–47].

Thyroid hormones inhibit the proliferation of adipocytes and stimulate their differentiation. They also regulate lipid metabolism by affecting the expression of lipolytic enzymes, increasing the consumption of oxygen, and altering the sensitivity of the tissue to other hormones. According to findings of some studies, the effect of thyroid hormones on the improvement of conditions caused by an increase in body fat percentage can be achieved through the body fat mass while increasing metabolism [45–52]. Viguerie et al. [49] showed that 19 genes of human white adipose tissue are regulated by thyroid hormones. These genes increase the proteins involved in signal transduction, lipid metabolism, apoptosis and inflammatory responses [49].

### Thyroid hormones and serum complement C3 levels

Our study showed a significant correlation between serum complement C3 and thyroid hormones. This finding of our study was consistent with findings of some previous studies [23, 53, 54], however we measured this relationship in normal weight obese individuals. Clare F et al. also observed the significant associations between thyroid hormones and markers of immune function including complement C3 in healthy individuals [20].

The thyroid gland and the immune system (intrinsic and acquired) are directly interrelated and affect each other [23, 32, 53]. Total T3 can modulate and regulate immune function through nuclear receptors and target genes, as well as non-genomic responses through membrane receptors [54, 55]. Moreover, the presence of TSH receptors on the surface of the immune cells indicates that this hormone stimulates the immune system, directly or via T3 and thyroxine (indirectly) [56, 57].

## Conclusion

An increase in body fat (despite of having a normal weight) can be accompanied by the changes in thyroid function and inflammatory markers such as complement C3 (early marker of metabolic disorders). If more fat is accumulated, these mechanisms will continue more intensively and will push the body towards metabolic disorders and pathological trends.

## Data Availability

Please contact corresponding author for data requests.

## Abbreviations

ASP: Acylation stimulating protein
BIA: Bioelectric impedance analyzer
BMI: Body mass index
FM: Fat mass
HPT: Hypothalamic-pituitary-thyroid
NWO: Normal weight obesity
REE: Resting energy expenditure
TSH: Thyroid-stimulating hormone
